# Population Density-Dependent Developmental Regulation in Migratory Locust

**DOI:** 10.3390/insects15060443

**Published:** 2024-06-11

**Authors:** Sifan Shen, Long Zhang, Liwei Zhang

**Affiliations:** 1 College of Grassland Science and Technology, China Agricultural University, Beijing 100193, China; 15501130702@163.com; 2 Institute of Plant Protection, Shandong Academy of Agricultural Sciences, Jinan 250100, China

**Keywords:** migratory locust, density, development, brain transcriptome, immunoglobulin

## Abstract

**Simple Summary:**

Our study reveals a density-dependent developmental pattern in locusts, which is associated with the classical ecdysone synthesis pathway. Additionally, analysis of the brain transcriptomic dataset uncovers varied metabolic, immune, and nutritional signals associated with density size. Specifically, *Immunoglobulin* (*IG*) emerges as a pivotal gene regulating density-dependent development. These regulatory molecules involved in density dependence present intriguing prospects for future investigation.

**Abstract:**

Insect development is intricately governed by hormonal signaling pathways, yet the pivotal upstream regulator that potentiates hormone activation remains largely elusive. The migratory locust, *Locusta migratoria*, exhibits population density-dependent phenotypic plasticity, encompassing traits such as flight capability, body coloration, and behavior. In this study, we elucidated a negative correlation between population density and ontogenetic development during the nymphal stage of locusts. We found that the level of density influences the developmental trajectory by modulating transcript abundance within the ecdysone signaling pathway, with knockdown of the prothoracicotropic hormone (PTTH) resulting in developmental delay. Transcriptomic analysis of locust brains across solitary and gregarious phases revealed significant differential expression of genes involved in various pathways, including protein synthesis, energy metabolism, hormonal regulation, and immunity. Notably, knockdown experiments targeting two energy regulators, adipokinetic hormone (AKH) and *insulin-like polypeptide 1* (*ilp1*), failed to elicit changes in the developmental process in solitary locusts. However, knockdown of *immunoglobulin* (*IG*) significantly shortened the developmental time in higher-density populations. Collectively, our findings underscore the regulatory role of population density in determining developmental duration and suggest that an immune-related gene contributes to the observed differences in developmental patterns.

## 1. Introduction

Insect metamorphosis represents a pivotal evolutionary event contributing significantly to the diversification and proliferation of arthropods, ultimately establishing insects as the most abundant, diverse, and widely distributed animal group on Earth. This transformative process is intricately regulated by various environmental stimuli, including temperature, humidity, and population density. Among the key regulatory factors, the antagonistic interplay between ecdysone and the juvenile hormone (JH) stands out as a fundamental role, orchestrating insect development and metamorphosis [1]. Ecdysone, synthesized and secreted by the prothoracic glands (PGs) under the control of the prothoracicotropic hormone (PTTH), drives larval molting, while JH, produced by glandular cells within the corpora allata (CA), maintains the larval state [2]. The dynamic interaction between JH and 20-hydroxyecdysone (20E) governs a myriad of physiological processes, including tissue apoptosis, remodeling, molting, and other developmental activities [1,2,3]. Low concentrations of 20E regulate growth, whereas high concentrations orchestrate the molting process in insect larvae [4]. In the process of metamorphosis, 20E induces programmed cell death (PCDI) and autophagy (PCDII), facilitating the transition from old larval tissues to newly formed adult tissues and safeguarding the midgut against infections [5]. Elevated JH levels during the larval stages ensure that the surge in 20E concentration leads to further larval stages and delays metamorphosis. However, as larvae approach their final instar, JH levels decline, allowing for the high 20E titer to instigate the metamorphic transition from larval to pupal or adult stages [6].

Collectively, larval metamorphosis is orchestrated by the intricate interplay of multiple hormonal pathways, with various hormones collaborating to regulate larval tissue growth until an appropriate size is reached for triggering the metamorphosis program [7,8]. *Kr-h1*, a C2H2 zinc-finger-type transcription factor, serves as an early inducible gene in the JH signaling pathway. Upon the knockdown of *Kr-h1* in either the fourth or fifth instar of *Blattella germanica* females, the length of the fifth instar in the ds*Kr-h1*-treated specimens is significantly longer than that for the controls. Subsequently, this *Kr-h1* suppression induces precocious adults after molting in the fifth instar [9]. Additionally, *Kr-h1* modulates metamorphosis by inhibiting the expression of ecdysone-inducible genes, such as *Broad* or *E93* [10,11]. The Halloween genes belong to cytochrome P450 superfamilies that play a crucial role in the 20E synthesis pathway, and they are reported to regulate insect development and metamorphosis. Silencing *LmSpookier*, a Halloween gene in migratory locusts, via RNA interference (RNAi) results in significant molting delays, molting failures, and wing development defects, which can be rescued by exogenous 20E injection [12]. Furthermore, JH has been implicated in mediating signal transduction cascades in the peripheral nervous system. The vitellogenin (Vg)-JH regulatory module regulates carbohydrate metabolism during the transition from nursing to foraging in worker bees, although it does not affect lipid metabolism [13]. In migratory locusts, the upregulation of JH induces a behavioral shift from attraction to repulsion towards volatiles emitted by gregarious locusts [14]. Similarly, JH and 20E are known to be controlled differently between solitary and gregarious nymphs in desert locusts to participate in the determination of the phase states. For example, solitary locusts have a higher JH titer in hemolymph, and manipulating the JH titer changes the phase state [15]. However, the understanding of the connections between the central nervous system, endocrine factors, and development remains incomplete in migratory locusts.

Insect development is intricately influenced by both internal physiological cues and external environmental factors. The perception of and response to environmental cues are critical for the developmental progression and survival of organisms, driving adaptive evolutionary processes [16,17]. In the context of insects, population density often exerts a negative regulatory effect on developmental processes, leading to delayed development, reduced body mass, and diminished survival rates [18,19,20,21,22,23,24]. The phenotypic alterations observed in response to crowded environments are commonly attributed to intensified competition for limited nutritional resources [25]. However, studies indicate that increasing food availability does not alleviate developmental delays and other phenotypic effects induced by high population density in insects such as *Lygus hesperus* [19]. This suggests that dietary restriction may not solely account for the stress induced by high population density. Despite these observations, there remains a dearth of molecular understanding regarding the precise mechanisms through which insect population density influences individual developmental processes. Further research is warranted to elucidate the molecular underpinnings of this phenomenon.

The migratory locust, *Locusta migratoria*, poses a significant threat to agricultural systems, displaying characteristic density-dependent phenotypic plasticity within its habitats [26]. Consequently, it serves as a valuable model organism for investigating the intertwined regulation of population density and intrinsic factors on insect development. In this study, we elucidated the influence of population density on locust development, highlighting the pivotal role of the ecdysone synthesis pathway in mediating this regulatory mechanism. Contrary to expectations, we detected no involvement of the AKH and insulin signaling in the brain in mediating the effects of population density on locust development. Additionally, we delineated multiple physiological signaling pathways potentially contributing to the density-dependent developmental plasticity in locusts. The *immunoglobulin* (*IG*) gene knockdown of immune-related pathways can significantly shorten the development time, shedding light on the intricate mechanisms governing this phenomenon.

## 2. Materials and Methods

### 2.1. Insects

Locusts were obtained from the Department of Entomology, China Agricultural University, and maintained in the laboratory at 30 ± 2 °C, 50 ± 10% RH, with a 16 h light/8 h dark photoperiod. The locusts were fed daily with fresh wheat.

### 2.2. Developmental Quantification

**Figure 1:**
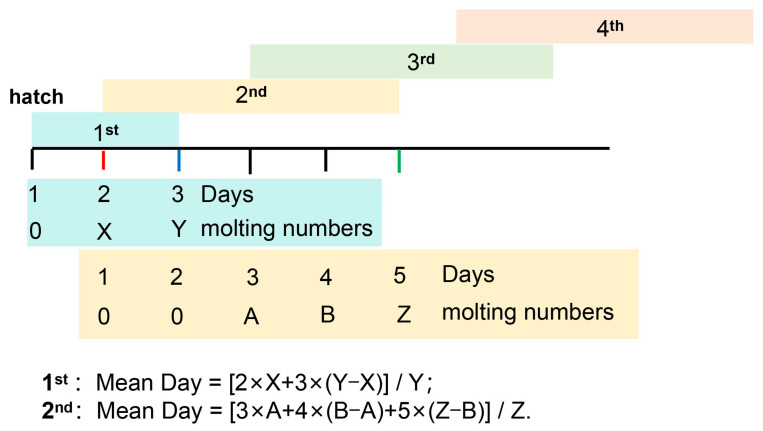
Statistical method of mean developmental time at a single instar. The developmental time of the 2nd instar represents the duration from the 2nd instar molting to the 3rd instar in all individuals. The red line represents the 1st individual molting to the 2nd instar, the blue line represents all individuals molting to the 2nd instar, and the green line represents all individuals molting to the 3rd instar.

Both gregarious (high-density) and solitary (single-rearing) migratory locusts hatched from gregarious eggs and the hatchlings were used to prepare gregarious and solitary locusts in the same generation. The gregarious groups were reared in large, well-ventilated cages (diameter: 13 cm × length: 40 cm), with a density of about 200 nymphs per cage. Solitary groups were kept individually in small cages (11 cm × 11 cm × 13 cm). The middle-density group contained five nymphs from gregarious hatchlings per cage (11 cm × 11 cm × 13 cm). We did not cross isolated-reared gregarious offspring to obtain typical solitary offspring as a second generation, since the current generation of isolated-reared gregarious offspring was sufficient to induce significant behavioral traits, as in typical multi-generation solitary locust, based on our open-arena assay. All the tested insects were fed with fresh wheat seedlings twice a day to avoid the negative effects of insufficient food on development. A cohort of at least 40 hatchlings from either the gregarious or solitary group was monitored to follow the development process. No cohort replicate was included in this study. We checked and recorded the developmental status of the tested nymphs between 8 and 10 p.m. every night until they molted to the fifth instar. The development time (days) required between the second and fourth instar was recorded and calculated using the following formula: [days_1 × A + days_2 × (B − A) + ∙∙∙ + days_n × (Z − Y)]/Z, where *days* means the accumulative days after the onset of development, and A-Z means the accumulative number of molted nymphs at 1 n days (see illustration in Figure 1). For instance, the development duration of the first nymph was calculated as Mean Day = [2 × X + 3 × (Y − X)]/Y, and the development duration of the second nymph was calculated as Mean Day = [3 × A + 4 × (B − A) + 5 × (Z − B)]/Z (Figure 1). The mean accumulative development days in each instar (between the second and the fourth instar) were calculated.

### 2.3. Real-Time Quantitative PCR

Brain tissues were taken from the second–third day of the fifth-instar nymphs. RNA was used for the quantification of the PTTH expression level. Prothoracic gland tissues were taken from the fifth-instar nymphs on days 1–6 for RNA extraction to quantify the dynamic changes in the expression levels of the 20E synthesis genes. The samples used in molecular experiments were equally mixed between the sexes. We used TRIzol Plus reagent (Ambion, Austin, TX, USA) to extract total RNA, and cDNA was synthesized from 1 μg of total RNA using the GoScript™ Reverse Transcription System kit (Promega, Madison, WI, USA). We mixed the Q-PCR reaction using the SuperReal PreMix Plus (SYBR Green) kit (Tiangen, Beijing, China): a 10 μL of 2× SuperReal PreMix Plus (SYBR Green) solution, 0.6 μL gene-specific primers (Supplementary Table S1), 5.8 μL of nuclease-free water, 1 μL of undiluted cDNA, and 2 µL of ROX. Reactions were performed using the ABI QuantStudio 6 Flex Real-Time PCR system (Thermo Fisher Scientific, Wilmington, NC, USA), with the following procedure: 95 °C, 15 min; 40 cycles (95 °C, 10 s; 60 °C, 30 s). The *rp49* gene was used as an internal reference. The relative expression levels of the target genes were calculated using the 2^−ΔΔCT^ method.

### 2.4. RNA Interference

The primers were designed with a T7 polymerase promoter sequence (TAATACGACTCACTATAGG) at the 5′ end (Supplementary Table S2) to amplify DNA templates for in vitro transcription to produce double-stranded RNA (dsRNA). Template DNA was purified with Wizard^®^ SV Gel and the PCR Clean-Up System (Promega, Madison, WI, USA). The dsRNA was synthesized and purified using the T7 RiboMAX™ Express RNAi System (Promega, United States) following the instructions provided. The dsRNA concentration was adjusted to 1 μg/μL and stored at −20 °C for later use. The RNAi assay was targeted for PTTH in gregarious locusts. The injection protocol was as follows: 3 μg of dsRNA was injected on the first day of the second instar, 4 μg of dsRNA was injected on the first day of the third instar, and 5 μg of dsRNA was injected on the first day of the fourth instar, with a total of 3 injections during the nymph development stage. RNAi assays for *AKH* and *ilp1* were performed in solitary nymphs following the same injection protocol in ds*PTTH*. The RNAi assay of *immunoglobulin* (*IG*) was performed in middle-density populations; when these nymphs molted to the third instar, 4 μg of dsRNA was injected on the first day of the third instar, and 5 μg of dsRNA was injected on the first day of the fourth instar, with a total of 2 injections during the nymph development stage. dsRNA was injected into the dorsal vessels of each locust using an IM-9B microinjector (Narishige, Japan) through the abdominal intersegmental membrane. A dsRNA microinjection of green fluorescent protein (GFP) was used as the control group. The injection time and amounts of nymphs in the control groups were consistent with those in the experimental groups.

### 2.5. Semi-Quantitative RT-PCR

On the second day of the fifth instar, we prepared an RNA sample from 8–10 brains pooled together, and we defined it as one independent biological repeat. The total RNA was extracted with TRIzol Plus reagent (Ambion, Austin, TX, USA), and then cDNA was synthesized from 1 μg of total RNA using the GoScript™ Reverse Transcription System kit (Promega). The RT-PCR reaction contained Taq DNA Polymerase (Tiangen, Beijing, China): 12.5 μL of the Mix solution, 1 μL gene-specific primers (Supplementary Table S1), 9.5 μL of nuclease-free water, 1 μL of cDNA. Reactions were performed using the Biometra PCR cycler, with the following procedure: 95 °C, 10 min; 25–35 cycles (95 °C, 10 s; 60 °C, 30 s). The same amount of the PCR product (GelRed staining) between groups was separated by 1.2% agarose gel electrophoresis and photographed with a gel imaging system.

### 2.6. RNA-seq and Bioinformatic Analysis

The total RNA was extracted using the TRIzol Plus reagent, and the integrity and purity of the total RNA were determined via the Agilent Bioanalyzer 2100 system (Agilent Technologies, Santa Clara, CA, USA) and NanoDrop2000 (Thermo Fisher Scientific, Wilmington, NC, USA). Samples of around 1 μg high-quality RNA were used to generate sequencing libraries, according to the NEBNext UltraTM RNA Library Prep Kit for Illumina (NEB, Ipswich, MA, USA). There were three biological repeats. Qubit 2.0 and Agilent 2100 were used to detect the concentration and insert size of the library. After the library was constructed, the inserts of the library were detected using the Qsep400 high-throughput analysis system, and then the effective concentration of the library (library effective concentration > 2 nM) was accurately quantified using the Q-PCR method to ensure the quality of the library. Then, PE150 mode sequencing was performed using a high-throughput sequencing platform. Bioinformatic analysis was performed using BMKCloud (www.biocloud.net (accessed on 12 November 2023)). Differential expression analysis of two samples was performed using DESeq2. Thresholds of FDR < 0.001 and Fold Change ≥ 2 were set for significantly differential expression. We used KOBAS software (https://bio.tools/kobas (accessed on 2 December 2023)) to test the statistical enrichment of differential expression genes in KEGG pathways. A principal component analysis plot, a volcano plot, and a heatmap plot were analyzed and plotted using Hiplot (https://hiplot.com.cn/cloud-tool/drawing-tool/list (accessed on 2 December 2023)).

### 2.7. Statistical Analysis

GraphPad Prism 8.0.2 software was used for statistical analysis and plotting. The Mann–Whitney test, Tukey’s multiple comparisons test, and nonparametric multiple *t*-test were used to analyze data from development experiments and Q-PCR. The thresholds for significance were as follows: * *p* < 0.05; ** *p* < 0.01; *** *p* < 0.001. Data are presented as the mean ± S.D.

## 3. Results

Population density is an important environmental factor affecting insect development and population growth, but its molecular basis is little understood. Migratory locusts exhibit population density-dependent phenotypic plasticity, which provides a good model to explore the effects of density on development. This study aims to reveal the underlying molecular mechanism by which population density affects development through multiple approaches.

### 3.1. High Density Restricts Locust Development

Large differences in developmental duration exist between phase stages in both migratory locusts [27] and desert locusts [8]. In order to ascertain the potential correlation between population density and developmental progression, we established a standardized experimental framework to investigate density-dependent developmental patterns (Figure 1).

**Figure 2:**
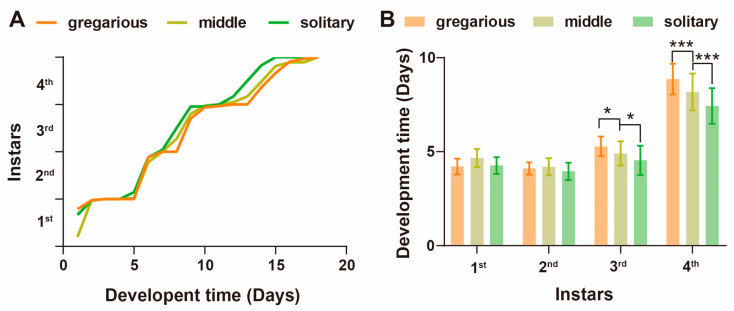
Differences in the development of locusts at different densities during the nymph stage. (**A**) Time-dependent developmental curve from the 1st to the 4th instar. The ordinate represents the proportion of individuals molting each day at each instar. (**B**) Cohort developmental duration at multiple instars. Cohort size = 40 ± 5. Tukey’s multiple comparisons test. *: *p* < 0.05; ***: *p* < 0.001. All data are presented as the mean ± S.D.

Through detailed characterization of the developmental durations across each instar (from first- to fourth-instar nymphs) within high-density gregarious, middle-density, and low-density solitary phases, we identified significant disparities in the developmental trajectory. Specifically, solitary locusts exhibited accelerated development relative to the gregarious phase and middle-density group (Figure 2A), with individuals experiencing lower population densities displaying notably shorter developmental periods during the later instars compared to those within higher-density populations (Figure 2B). These findings underscore the presence of density-dependent regulatory mechanisms governing the developmental processes across locust phases.

### 3.2. Ecdysone Synthesis Signaling Is Up-Regulated under Low Density

**Figure 3:**
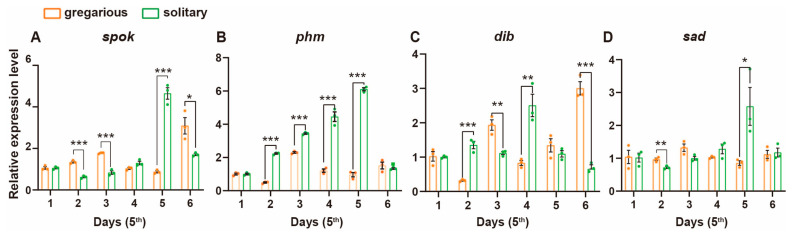
Quantitative real time PCR (Q-PCR) validation of 20E synthesis key genes in fifth nymphs. The abscissa indicates the development time after molting to the 5th instar. Orange: gregarious groups, green: solitary groups. Each dot represents a biological replicate. Reference gene: *rp49*. *n* = 3. Nonparametric multiple *t*-test. *: *p* < 0.05, **: *p* < 0.01, ***: *p* < 0.001. All data are presented as the mean ± S.D.

The titer of 20-hydroxyecdysone (20E) escalates preceding molting and subsequently declines following the formation of a new epidermis, thereby facilitating larval development [28,29,30,31]. The Halloween gene belongs to cytochrome P450 superfamilies that play a key role in the ecdysone synthesis pathway, including CYP307A1 (*spook*), CYP306A1 (*phantom*), CYP302A1 (*disembodied*), CYP315A1 (*shadow*), and CYP314A1 (*shade*). In turn, 20E is catalyzed by the gradual synthesis of these genes [32,33]. To investigate whether the signaling pathways responsible for 20E synthesis are modulated under differing population densities, we conducted a quantitative real-time PCR to profile the transcript abundance across the signaling cascade. Our analysis focused on four key Halloween genes expressed within the prothoracic gland throughout the fifth instar (Figure 3). The relative expression levels of *spook* (*spok*), *phantom* (*phm*), *disembodied* (*dib*), and *shadow* (*sad*) exhibited a notable increase towards the late fifth instar, peaking one day prior to molting. Intriguingly, the transcriptional activity of all genes, except for *dib*, was markedly potentiated in solitary locusts on the day preceding molting. In contrast, individuals within the gregarious phase maintained lower levels of gene transcription throughout the developmental stage. These findings suggest a form of putative hormonal regulation involved in density-dependent developmental plasticity, particularly through the modulation of ecdysone signaling pathways.

### 3.3. PTTH Is Not Directly Involved in Density-Regulated Developmental Pathways

**Figure 4:**
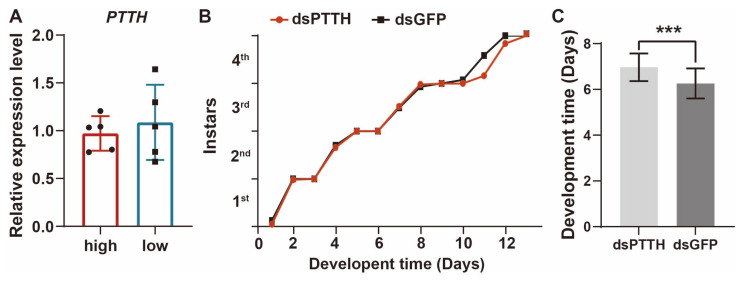
Effects of *PTTH* on development. (**A**) Comparison of *PTTH* expression levels in the brains of solitary and gregarious locusts. Each dot represents a biological replicate. (**B**) Nymph development trend from the 1st to the 5th instar after knockdown the *PTTH* expression level. The ordinate represents the proportion of individuals molting each day at each instar. (**C**). Comparison of 4th instar developmental time after injection of ds*PTTH* and dsGFP. *n* = 40. Mann–Whitney test. *** *p* < 0.001. All data are presented as the mean ± S.D.

**Figure 5:**
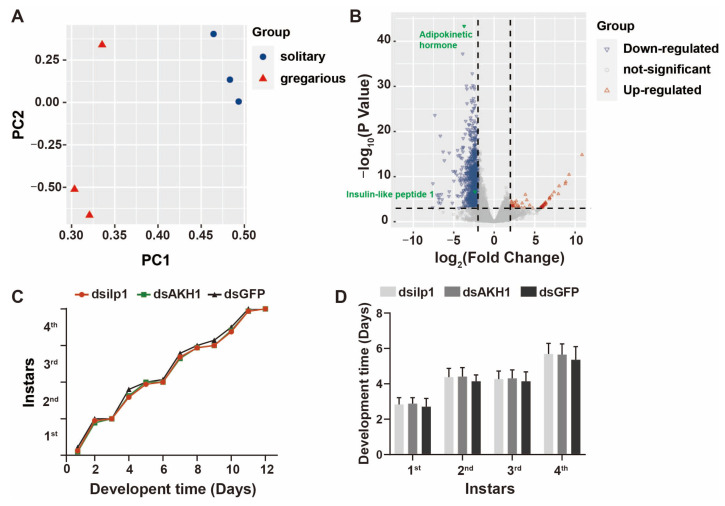
Differential developmental transcription patterns in the brains of different densities. (**A**) Principal component analysis of transcript samples from solitary and gregarious brains. (**B**) Volcano diagram of DEGs. The dotted line represents the screening range of DEGs: *p*-value < 0.001, Fold Change ≥ 2. Blue: down-regulated genes, Red: up-regulated genes, Green: represented genes. (**C**,**D**) Effects of knockdown the AKH/ilp1 on the development of locusts. *n* = 24. Nonparametric multiple *t*-test. All data are presented as the mean ± S.D.

The prothoracicotropic hormone (PTTH) is synthesized by neurosecretory cells within the insect brain and subsequently released into the hemolymph, where it plays a crucial role in regulating ecdysone synthesis within the prothoracic glands [34,35]. Alterations in PTTH titers within the insect brain and hemolymph have been linked to the induction of diapause in insects [28,36]. Our investigation sought to elucidate whether the transcriptional dynamics of PTTH are implicated in generating distinct levels of 20-hydroxyecdysone (20E) signaling between different phases. Quantitative PCR (Q-PCR) analysis revealed no significant differential expression of *PTTH* transcripts within the brain under varying population densities (Figure 4A). This expression consistency was also demonstrated in the transcriptomic analysis (Figure 5B). However, the knockdown of *PTTH* expression significantly extended the developmental period required during the fourth instar of the dense population (Figure 4B,C). It is worth noting that *PTTH* transcripts were successfully suppressed in the brains (Figure S1A). This observation prompted our inquiry into whether density-induced disparities in ecdysone synthesis are directly regulated by the transcriptional mechanisms governing *PTTH*. It is conceivable that post-transcriptional modifications or other non-mRNA-mediated mechanisms may contribute to *PTTH*-mediated developmental plasticity across varying population densities.

### 3.4. Differential Developmental Transcription Patterns in the Brains of Different Phases

The central nervous system, particularly the brain, plays a pivotal role in orchestrating both the behavioral repertoire and developmental processes of insects [37,38]. The completion of locust genome sequencing has greatly promoted the study of functional genomics by providing us with a well-annotated database [39,40,41]. In our study, we sought to explore the potential link between brain molecular profiles and density-dependent developmental plasticity through the transcriptomic sequencing of locust brains under varying population densities. Principal component analysis (PCA) revealed distinct clustering patterns, indicative of clear segregation between the solitary and gregarious phases (Figure 5A). Subsequent volcano plot analysis unveiled numerous differentially expressed genes (DEGs) exhibiting phase-specific expression preferences, with a notable predominance of downregulated genes among the DEGs (Figure 5B). Notably, the adipokinetic hormone (AKH) emerged as the most prominently downregulated gene in the gregarious phase, suggesting a potential regulatory role in density-dependent developmental processes. The solitary-biased expression of AKH was also demonstrated with relative quantification (Figure S1B).

The AKH represents a crucial endocrine neuropeptide in insects, pivotal for the regulation of energy homeostasis by facilitating the breakdown of nutrient reserves within fat bodies, thereby sustaining high-energy-demanding physiological activities [42,43,44]. Given previous reports indicating disparities in food intake and energy utilization between solitary and gregarious phases [27], we hypothesized that distinct strategies in energy processing and utilization might underlie the density-dependent developmental regulation mediated by AKH. However, the RNA interference (RNAi)-mediated suppression of *AKH* in solitary locusts did not result in a deceleration in the developmental process (Figure 5C,D). Subsequently, we targeted another energy-related gene, insulin-like polypeptide 1 (ilp1), which exhibited significant downregulation in the solitary phase (Figure 5B and Figure S1B). Notably, the knockdown of ilp1 transcripts in solitary locusts similarly failed to exert any discernible effects on developmental progression (Figure 5C,D). It is worth noting that both AKH and ilp1 transcripts were successfully suppressed in the brains (Figure S1A). These findings suggest that the downregulation of both *AKH* and *ilp1* may represent physiological consequences arising from developmental divergence rather than serving as causal initiators of density-dependent developmental regulation.

### 3.5. Multiple Molecular Pathways Are Changed in Brains between Density Sizes

**Figure 6:**
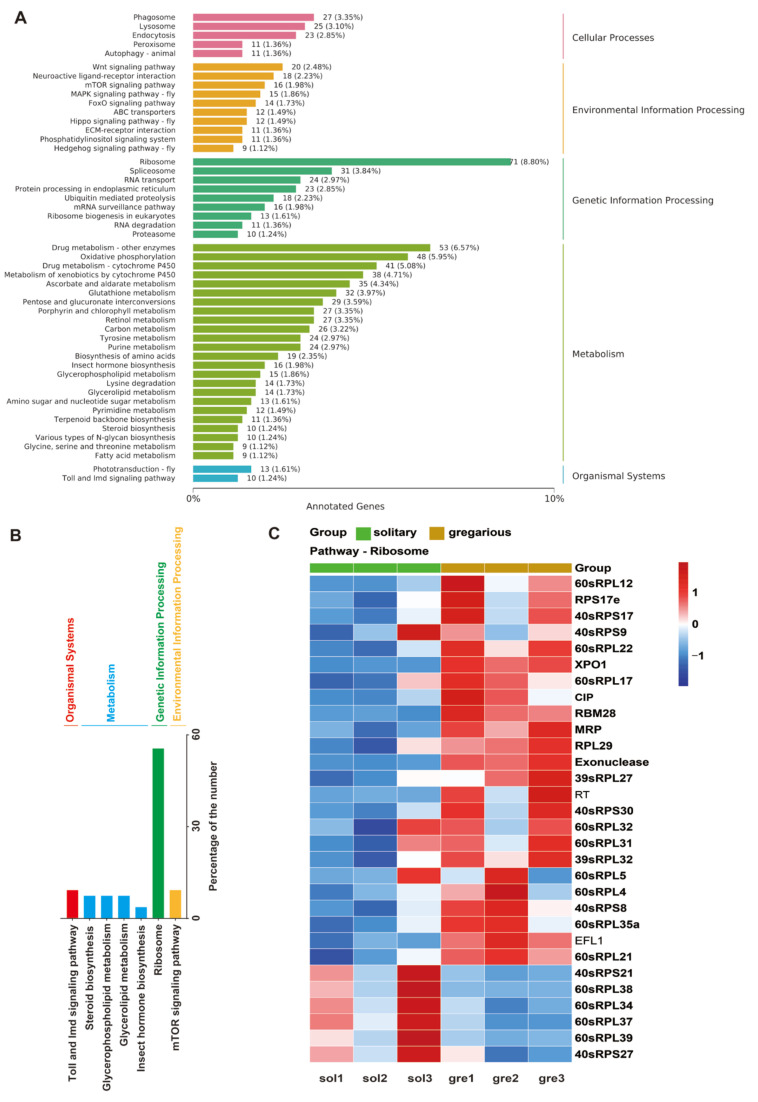
(**A**) Kyoto Encyclopedia of Genes and Genomes (KEGG) pathway classification of DEGs between solitary and gregarious locusts. (**B**) The representative pathways in KEGG terms. (**C**) Heatmap of DEGs in the ribosome pathway. sol: solitary, gre: gregarious. The expression levels of DEGs are highlighted in red (up-regulated) and blue (down-regulated).

**Figure 7:**
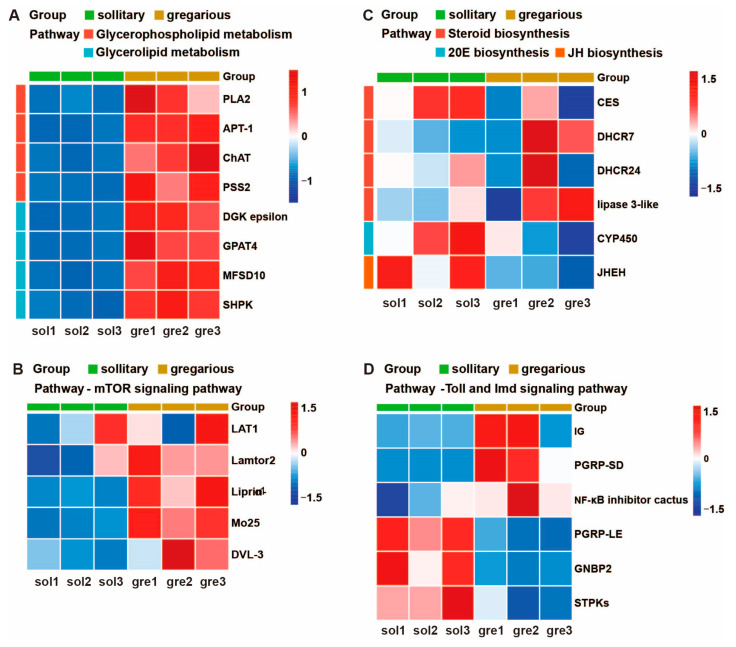
Heatmap of DEGs in the (**A**) glycerophospholipid and glycerolipid metabolism pathway, (**B**) mTOR signaling pathway, (**C**) steriod biosynthesis, 20E and IH biosynthesis pathway, and (**D**) Toll and Imd signaling pathway. The expression levels of DEGs are highlighted in red (up-regulated) and blue (down-regulated).

To comprehensively delineate the transcriptomic disparities between solitary and gregarious locusts, we subjected a total of 806 differentially expressed genes (DEGs) to rigorous analysis and categorization into 50 distinct subcategories based on Kyoto Encyclopedia of Genes and Genomes (KEGG) signaling pathways (Figure 6A). Several subcategories, notably immunity, steroid and hormone biosynthesis, ribosome, metabolic processes, and mechanistic target of rapamycin (mTOR) signaling, exhibiting a significant abundance of DEGs, were prioritized for in-depth scrutiny (Figure 6B). These pathways have been previously implicated in locust polyphenism. Among the selected subcategories, the ribosomal pathway stood out with the highest proportion (76%) of DEGs (Figure 6B). Notably, a majority of ribosomal genes exhibited upregulation in the gregarious phase (Figure 6C), suggesting substantial alterations in protein synthesis dynamics between the two phases, as reported previously [45]. Furthermore, approximately 70% of the DEGs were associated with metabolic processes, underscoring pronounced disparities in energy utilization and metabolic patterns between the solitary and gregarious phases (Figure 6A). Given the pivotal role of energy metabolism in developmental speed, particularly in facilitating rapid growth necessitating additional energy supply, we scrutinized the pathways related to glycerophospholipid and glycerolipid metabolism. Within these pathways, eight genes including *phospholipase A2* (*PLA2*), *acyl-protein thioesterase 1* (*APT1*), *choline acetyltransferase* (*ChAT*), *phosphatidylserine synthase 2* (*PSS2*), *diacylglycerol kinase epsilon* (*DGK epsilon*), *glycerol-3-phosphate acyltransferase 4* (*GPAT4*), *major facilitator superfamily domain-containing protein 10* (*MFSD10*), and *sedoheptulokinase* (*SHPK*) were notably upregulated (Figure 7A). Moreover, the mTOR signaling pathway, known to heavily promote cell growth and proliferation by upregulating protein and lipid synthesis while bolstering cellular metabolism and downregulating apoptotic pathways, displayed upregulation in five genes, including the *large neutral amino acids transporter* small subunit 1 (*LAT1*), *Lamtor2*, *liprin-alpha-1* (*liprin-α1*), *Mo25*, and *dishevelled segment polarity protein 3* (*Dvl3*) (Figure 7B). Collectively, the upregulation of multiple energy metabolism signaling genes in transcripts suggests enhanced energy production in gregarious locusts, illuminating a potential mechanism underlying their density-dependent developmental regulation.

The hormonal system plays a critical role in insect development and also governs phase-specific olfactory behaviors in locusts. Within the steroid biosynthesis pathway, we observed significant alterations in gene expression profiles. Specifically, *carboxylesterase* (*CES*) exhibited downregulation, while *lipase 3*-like, *7-dehydrocholesterol reductase* (*DHCR7*), and *delta (24)-sterol reductase*-like (*DHCR24*) were upregulated (Figure 7C). Moreover, *cytochrome P450* (*CYP450*) involved in the 20E synthesis pathway and *juvenile hormone epoxide hydrolase* (*JHEH*) involved in the JH synthesis pathway were markedly suppressed in gregarious locusts (Figure 7C). Additionally, previous studies have elucidated the phenomenon of density-dependent prophylaxis (DDP) in locusts, wherein individual immunity increases with larger group sizes or densities [46,47]. KEGG classification revealed that six differentially expressed genes (DEGs) were categorized into insect innate immune pathways (Toll and Imd signaling), encompassing three upregulated genes, *immunoglobulin* (*IG*), *peptidoglycan-recognition protein SD* (*PGRP-SD*), and *NF-kappa-B inhibitor cactus*, along with three downregulated genes: *peptidoglycan-recognition protein LE* (*PGRP-LE*), *GNBP2*, and *serine/threonine-protein kinase* (*STPKs*) (Figure 7D). Furthermore, more than 80% of genes associated with cellular processes were classified under phagosomes and lysosomes, common cellular structures implicated in immune processing (Figure 6A). Taken together, these findings suggest that alterations in population density induce shifts in energy distribution and metabolic patterns in locusts, with heightened energy allocation toward immune function potentially contributing to prolonged development times in high-density populations and, conversely, expedited development in low-density populations.

**Figure 8:**
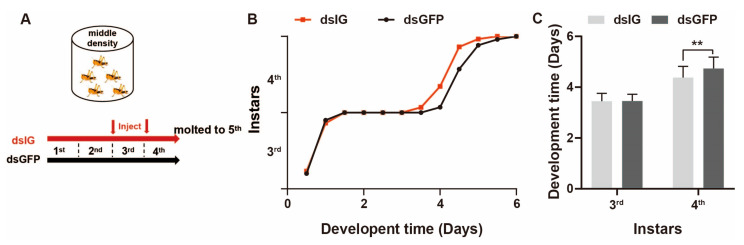
Knocking down *IG* accelerates nymphal development in the middle-density group. (**A**) RNAi assay setup for *IG* in the middle-density group. (**B**). Nymph developmental trajectory from the 3rd to the 5th instar after knockdown *IG*. The ordinate represents the proportion of individuals molting each day at each instar. (**C**) Comparison of 3rd and 4th instar developmental times after injection of ds*IG* and ds*GFP*. *n* = 30 in each group. Mann–Whitney test. ** *p* < 0.01. All data are presented as the mean ± S.D.

To illuminate the molecular regulators involved in density-dependent developmental plasticity, we needed to remove the potent effects from phase change. We found one gene, *immunoglobulin* (*IG*), that was upregulated in both the middle-density and gregarious groups in comparison to solitary locusts (Figure 5B). We knocked down the expression of *IG* in middle-density populations using RNAi and observed the effects on development (Figure 8A). This indicated that after the second injection, the developmental trajectory of ds*IG*-injected locusts separated from that of the ds*GFP* injection group (Figure 8B). The development time at the fourth instar after ds*IG* injection was significantly shortened (Figure 8C and Figure S1A). These results suggest that the higher expression of *IG* in the middle-density group extended the development duration compared with solitary locusts.

## 4. Discussion

Sensing and responding to environmental cues are integral for insect development and survival, driving adaptive evolution [2]. In this study, we elucidated that higher population density correlates with a slower developmental rate, yet the specific signals within dense populations that influence developmental processes remain enigmatic. Several hypotheses have been proposed to explain the effects of density cues on insect development, encompassing tactile interferences, chemical information stimuli, and pressure on food distribution [25,48,49]. For instance, larvae of *Culex quinquefasciatus* adjust to crowded conditions by decelerating development, while increasing the specific surface area to minimize the physical contact between larvae significantly mitigates the effects of crowding [49]. Notably, tactile stimuli play a pivotal role in the phase transition process of the desert locust [50], suggesting that a similar mechanism may operate in migratory locusts, where tactile stimuli likely represent a key factor influencing development. In addition to tactile cues, olfactory cues emerge as potential mediators in developmental regulation, as numerous studies demonstrate the association between phase changes and volatile compounds [51,52]. Identifying the key sensory cues governing density-dependent developmental plasticity in migratory locusts is of particular interest. Moreover, elucidating the intricate process by which sensory activation is transduced into final developmental control entails unraveling the complex neuroendocrine signaling pathways that likely play critical roles in signal transduction.

Various phenotypic changes observed in immature insects exposed to crowded environments have traditionally been attributed to the intensified competition for limited nutritional resources [25]. However, empirical evidence challenges this notion, as demonstrated by the inability of increased food availability to alleviate the developmental delays and other phenotypic effects induced by high population density in *Lygus hesperus* [19]. This suggests that dietary restriction may not be the primary stressor associated with high population density. In the developmental assessment conducted in this study, the nymphal stage of the migratory locust exhibited a negative regulatory response to population density, despite ample food provision ensuring no nutrient limitations. Intriguingly, although the high-density group experienced no dietary restrictions, the amplification of negative effects due to crowded conditions, coupled with the abundance of food resources, may modulate individual development through its influence on the neuroendocrine system.

In *Schistocerca gregaria*, gregarious individuals typically undergo five molts, while solitary individuals tend to experience an additional molt starting from the fourth instar, resulting in six nymphal molts [53,54,55]. This additional molting process contributes to the development of sexual size dimorphism (SSD) [56]. Generally, females exhibit larger sizes than males, resulting in a phenomenon known as female-biased SSD. The catch-up growth strategy is proposed to explain the occurrence of additional molting in solitary populations of the desert locust, as solitary hatchlings are considerably smaller than their gregarious counterparts [57]. However, in our experiment, we did not observe the phenomenon of additional molting. This lack of observation is not unexpected, given the contrasting developmental and phase-related traits between desert locusts and migratory locusts. For instance, solitary-reared desert locusts subjected to crowding with 10 crowd-reared nymphs exhibited gregarious-like behavioral changes after just 4 h of crowding [58]. This rapid transition from solitary to gregarious phases contrasts sharply with the behavior of migratory locusts, which do not display significant behavioral changes indicative of gregarization until after 32 h of crowding [51]. This substantial difference between the two species likely stems from differences in their developmental processes. *Schistocerca gregaria* exhibits distinct patterns of nymphal development between phases, with gregarious hatchlings growing faster and emerging as larger adults compared to solitary hatchlings under isolated conditions [59]. Interestingly, in our study, we observed that solitary nymphs of the migratory locust grew faster and had shorter development durations than gregarious nymphs, which is contrary to the findings in desert locusts [59].

The quantitative results of *PTTH* mRNA levels in the brain showed that there was no significant difference between high and low density. The main function of *PTTH* is to regulate the synthesis of 20E. *PTTH* mutation downregulates ecdysone synthesis genes and decreases ecdysone titers, resulting in defects, such as delayed larval metamorphosis, critical weight gain, and unbalanced organ development [60,61]. We found developmental delays by knocking down the expression level of *PTTH*, but the consistent expression of *PTTH* at different densities indicated that other signaling pathways are involved in the regulation of the 20E signaling pathway, like the antimetamorphic factor *Kr-h1*. The insect *dopamine*/*ecdysteroid receptor* (*DopEcR*) plays an important role in insects’ responses to stressors, including crowding stress, so we focused on the effect of density on ecdysone [62]. The relationship of the cross-regulation between hormones and environmental factors, like density and developmental processes, deserves further attention.

Migratory locusts exhibit slower developmental rates in response to increased population density. In scenarios where food availability is not limited by nutrient ion constraints, high-density populations allocate less energy towards developmental processes compared to solitary individuals. This prompts the following inquiry: where is the surplus energy allocated, and how do gregarious locusts benefit from delayed development? One plausible explanation is that gregarious locusts conserve energy from delayed development and redirect it towards bolstering their immune defenses, particularly given the heightened risk of pathogen infections within dense populations. Indeed, empirical evidence has demonstrated that gregarious locusts exhibit greater resistance to pathogens compared to solitary phases [63,64]. Notably, locusts possess density-dependent prophylaxis (DDP), wherein high-density populations display enhanced resistance to disease, while low-density populations are more susceptible to pathogenic fungal infections [46,47]. Transcriptomic analyses have revealed significant alterations in genes associated with the Toll and Imd signaling pathways, underscoring the intricate interplay between density-dependent developmental strategies and immune responses. Decreased IG expression in the brain leads to shortened development, although it is unclear how density regulates the brain and thus affects the relationship between development and immunity. In *Drosophila*, a novel immune modulator, *IM33*, sustains the lifespan by controlling the gut microbiota, and the expression of *PRGP-LE* in brain insulin-producing cells can modulate sleep behavior and regulate the lifespan [65]. At present, there is no good explanation for the role of *IG* in density-regulated development and immunity. Consequently, a pivotal question emerges: how do gregarious locusts finely balance the energy allocation between developmental processes and immunity in response to changes in population density? Exploring the regulatory molecules involved in this dynamic energy balance presents an intriguing avenue for future research.

## Data Availability

The data presented in this study are available on request from the corresponding author due to privacy reasons.

## Supplementary Materials

The following supporting information can be downloaded at: https://www.mdpi.com/article/10.3390/insects15060443/s1, Figure S1. RNAi efficiency confirmation; Table S1. The primers for Q-PCR; Table S2. The primers for dsRNA preparation.
