# Supplementary material for: Population Density-Dependent Developmental Regulation in Migratory Locust
Source: Insects. 2024 Jun 11;15(6):443. doi: 10.3390/insects15060443 (PMC11203946; doi:10.3390/insects15060443)
Supplement: Supplementary file 1 [file insects-15-00443-s001.zip › insects-3007602-supplementary.pdf]

Supplementary Materials

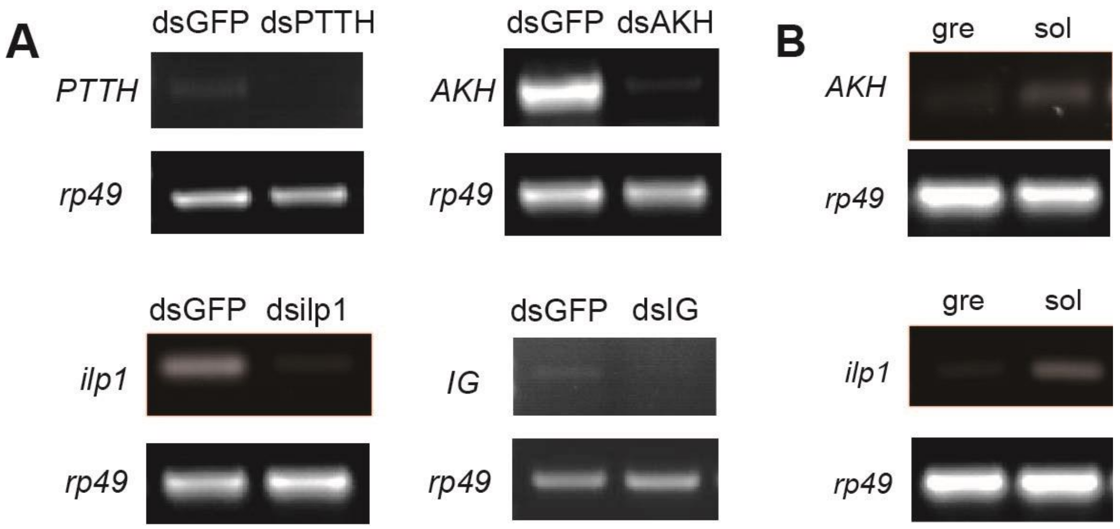

Figure S1. RNAi efficiency confirmation. A. Semi-quantitative RT-PCR showed that RNAi specifically down-regulated the expression level of *PTTH*, *AKH*, *ilp1* and *IG*. B. Semi-quantitative RT-PCR showed relative quantification of *AKH* and *ilp1* transcripts between gregarious and solitary brains.

Table S1. The primers for RT-PCR and RT-qPCR.

Gene ID	Forward Primer (5' to 3')	Reverse Primer (5' to 3')
PTTH	AGTACAACGCCGAGAAGCT	AAGCAGTGTGGTCATCTGTG
AKH (LOCMI17565)	CGACTTCGCAGACCCCTA	TTGGAACACCCAGACATCTTC
ilp1 (LOCMI16379)	GCGAGAAGCTCTCCAACG	GCTCCAGTAGTTGTCTTCAG
IG (LOCMI13143)	CATTCGCCGATCATCTGGAA	AGGGAGTGAGCAGCAGAAAT

Table S2. The primers for dsRNA preparation.

Gene ID	Forward Primer (5' to 3')	Reverse Primer (5' to 3')
PTTH	AGCACGTCCATGACGCACT	GCAGCTTCTCGGCGTTGTAC
AKH (LOCMI17565)	ATGGTGCAGCGTTGCGCCCTGG TGG	GTTGGAACACCCAGACATCTT CCTG
ilp1 (LOCMI16379)	CCGACCTGTTCCTCCTGTC	GGCAGCACTCGTCGAAGA
IG (LOCMI13143)	CATTCGCCGATCATCTGGAA	AGGGAGTGAGCAGCAGAAAT

Note: The T7 promoter (GGATCCTAATACGACTCACTATAGG) is located at the 5' end of the primer sequence.
